# Yao syndrome: Cyclical folliculitis, fevers, and abdominal pain

**DOI:** 10.1016/j.jdcr.2023.01.039

**Published:** 2023-03-12

**Authors:** Ilhan Esse, Colin Kincaid, Luke Horton, Justin D. Arnold, Natasha A. Mesinkovska

**Affiliations:** Department of Dermatology, University of California, Irvine, California

**Keywords:** Blau syndrome, mastocytosis, NOD2, Yao syndrome

## Introduction

The nucleotide-binding oligomerization domain 2 (NOD2) is a prominent member of the nucleotide-binding domain, leucine-rich repeat containing protein family of intracellular sensors of pathogen-associated molecular patterns.^1^ It is found in many immune system cells, including monocytes, macrophages, and dendritic cells. It mainly recognizes muramyl dipeptide, which is a component present in bacterial cell walls, which activates the nuclear factor kappa-light-chain-enhancer of activated B cells protein and leads to an innate immune response.^2^ Mutations in NOD2 are associated with a group of systemic autoinflammatory diseases, which are characterized by unprovoked episodes of inflammation with a benign autoimmune workup.^3^ These include Blau syndrome (triad of granulomatous dermatitis, uveitis, and arthritis), sarcoidosis, and Crohn’s disease. A more recent addition, Yao syndrome, formerly known as NOD2-associated autoinflammatory disease, presents with erythematous plaques and patches, periodic fevers, myalgia, gastrointestinal (GI), and sicca-like symptoms.

## Case report

**Fig 1:**
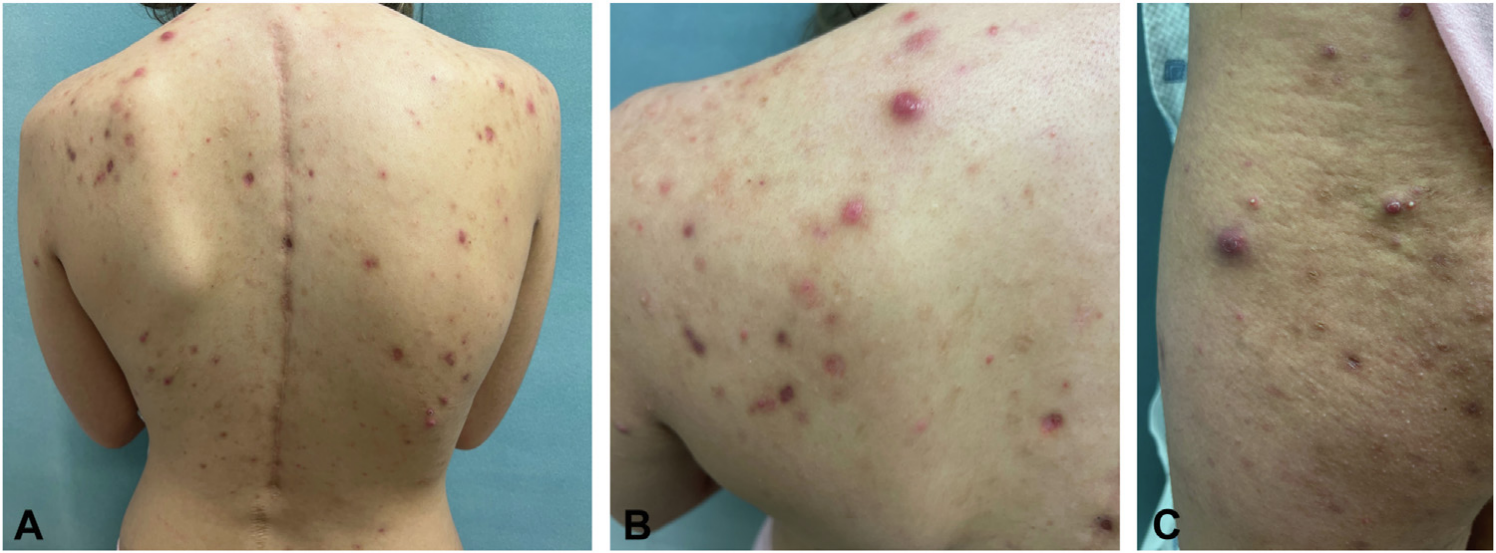
Skin findings of our patient with NOD2 mutation. **A, B** Erythematous papules and cystic nodules on the back. **C,** Cystic nodules extending from buttocks to lateral aspect of the thigh.

**Fig 2:**
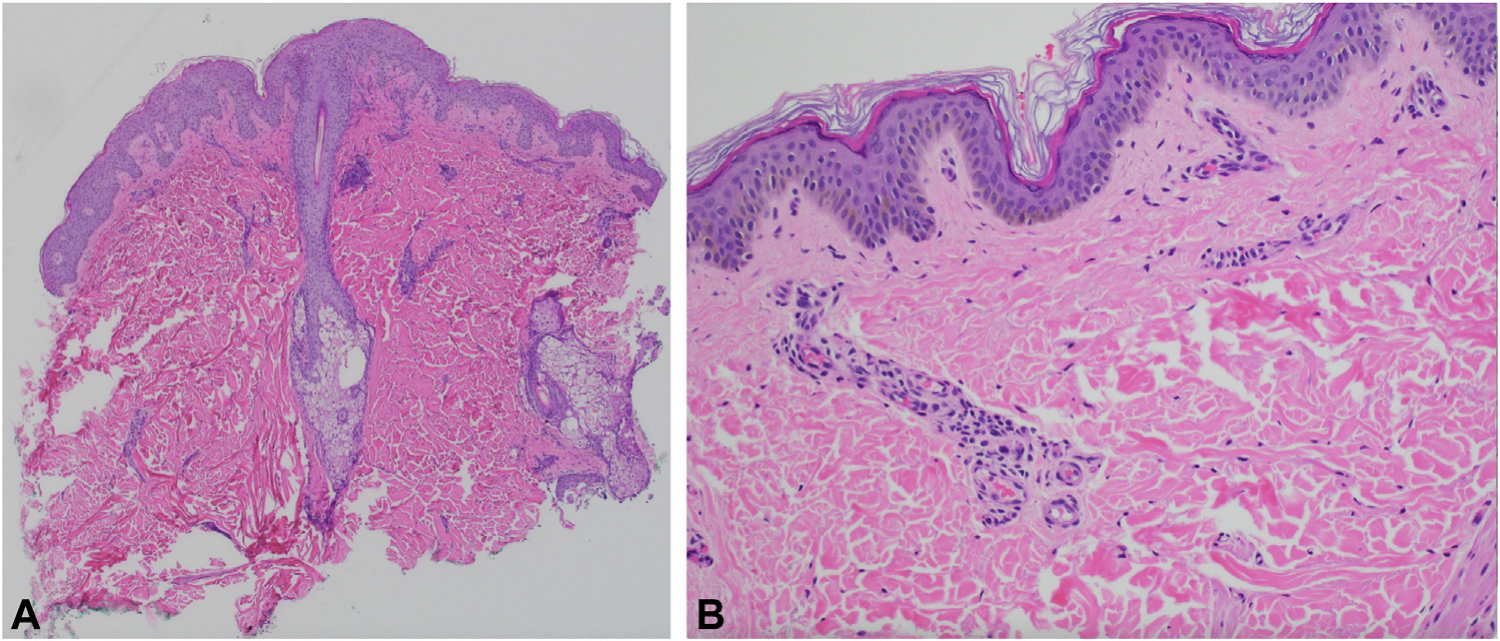
Histopathologic findings of inflamed nodule on the back and erythematous plaque on the lower extremity. **A,** Punch biopsy sample image of the lower back showing numerous neutrophils within the hair follicle, which is consistent with a suppurative folliculitis. **B,** Punch biopsy sample image of the left medial thigh with sparse mixed lymphocytic and neutrophilic perivascular inflammation (**A** and **B,** Hematoxylin-eosin stain; original magnifications: **A,** ×10; **B,** ×40.)

A 16-year-old woman presented to dermatology reporting 3 years of recurrent pruritic erythematous plaques and patches accompanied by cyclic fevers, flushing, joint pain, and GI symptoms, including abdominal pain and diarrhea. Before presentation, a comprehensive workup at an outside dermatologic facility diagnosed her with cutaneous mastocytosis without a biopsy analysis. However, despite years of appropriate anti–mast cell therapy, her symptoms persisted. On examination, she presented with well-demarcated, slightly erythematous plaques and patches on the extremities and buttocks (not depicted here). There were also numerous scattered, tender, erythematous cystic nodules on the inframammary folds, back, inner thighs, and buttocks areas (Fig 1). Three punch biopsies were performed on the inflamed nodules on her back and the erythematous plaques on her extremities and the samples were sent for histopathologic examination. Tissue section staining images of the back nodule revealed a suppurative folliculitis, whereas images of the extremity plaques demonstrated mixed lymphocytic and neutrophilic perivascular inflammation. There was no histologic evidence of cutaneous mastocytosis (Fig 2).

Given these inconclusive findings, she underwent an extensive autoimmune workup, investigating her thyroid markers; antinuclear antibodies; and C3, C5, IgG, IgA, and IgM levels, which were unremarkable. A primary immunodeficiency panel test performed by Invitae revealed a heterozygous c.3019dup (p.Leu1007Profs∗2) mutation on exon 11 of the *NOD2* gene. The same mutation was also found in her asymptomatic mother. The patient was followed up at our clinic biannually, during which time she had periodic skin flares every 4 to 8 weeks consisting of erythematous patches and plaques, cystic nodular eruptions, fevers, and abdominal pain. During these flares, treatment with acetaminophen 500 mg twice daily helped manage her fever and pain. Initial treatment with systemic glucocorticoids and sulfasalazine was inadequate in alleviating her cutaneous symptoms. We later considered treatment with a tumor necrosis factor-alpha inhibitor. Given the patient’s GI symptoms and the association of Crohn’s disease with NOD2 mutations, inflammatory bowel disease was excluded before pursuing a diagnosis of Yao syndrome. Colonoscopy was performed, the results of which revealed no signs of inflammatory bowel disease. Considering her constellation of symptoms, including fever, various cutaneous findings, GI symptoms, and arthralgias in the setting of a negative autoimmune workup and a known NOD2 mutation, we suspected that her presentation was consistent with Yao syndrome. The patient gave consent for her photographs and medical information to be published in print and online, with the understanding that this information may be publicly available.

## Discussion

Here, we present the case of a young woman with longstanding episodic dermatitis and folliculitis ultimately found to harbor a mutation in *NOD2* with a positive family history. The patient previously received a clinical diagnosis of cutaneous mastocytosis, which requires 1 major skin criterion (polymorphic, maculopapular reddish-brown lesions) and 2 minor criteria (skin biopsy results revealing considerable mast cells or activating receptor tyrosine kinase mutation) for diagnosis, none of which were demonstrated by our patient. This combination of findings led us to a diagnosis favoring Yao syndrome.^4^

Yao syndrome, which is a form of systemic autoinflammatory disease, is characterized by periodic fevers, erythematous plaques/patches, myalgia, GI, and sicca-like symptoms.^3^ Yao syndrome has been recently categorized as a genetically transitional disease, which is a genetic disease status between monogenic and polygenic diseases in which a mutation is required but is insufficient to cause disease.^5^ Most cases occur sporadically, typically presenting in adulthood. The most commonly described cutaneous findings are erythematous plaques and patches with various pathologic findings, including mixed lymphocytic and neutrophilic infiltrates, spongiotic dermatitis, and granulomatous changes.^3^ A patient can receive a diagnosis of Yao syndrome if 2 major criteria (≥2 periodic flares, recurrent fevers, or dermatitis), at least 1 minor criterion (arthralgia, abdominal pain/GI symptoms, sicca-like symptoms, or pericarditis/pleuritis), and the molecular criterion (NOD2 or R702W mutation), as well as exclusion criteria (negative autoimmune workup and exclusion of other autoinflammatory diseases) are fulfilled (Table I).^6^

**Table I:** The diagnostic criteria for Yao syndrome^5^

Clinical criteria	Description
Major	• Periodic occurrence ≥ 2 flares∗ • Recurrent fevers∗ • Dermatitis∗
Minor	• Arthralgia (oligo- or polyarthralgia/inflammatory arthritis, or distal extremity swelling)∗ • GI symptoms (abdominal pain or diarrhea)∗ • Sicca-like symptoms • Pericarditis • Pleuritis
Molecular Criterion	• NOD2∗ or R702W mutation
Exclusion criteria	• Negative autoimmune workup∗ • Exclusion of other autoinflammatory diseases∗

Yao syndrome is diagnosed if 2 major criteria, at least 1 minor criterion, the molecular criterion, and exclusion criteria are fulfilled.

*GI*, Gastrointestinal; *NOD2*, nucleotide-binding oligomerization domain 2.

∗ Criteria met by the patient in this case report.

Yao syndrome was first described in 2011 in a cohort of patients who presented with clinical features resembling Blau syndrome but lacked the classic triad seen in this condition (granulomatous dermatitis, uveitis, and arthritis) and who only developed findings in adulthood.^7^ The inflammatory polyarthritis seen in the patients with Yao syndrome lacked any joint deformities, such as camptodactyly, which is a common feature reported in Blau syndrome. Additionally, this cohort showed predominantly nongranulomatous dermatitis and no signs of uveitis, which is seen in at least two-thirds of patients with Blau syndrome.^7^ Other common NOD2-associated conditions, such as Crohn’s disease and sarcoidosis, could also be considered in affected patients; however, periodic fevers, sicca-like symptoms, and nongranulomatous dermatitis are uncommon in both. With respect to therapeutic management, many patients with Yao syndrome respond well to glucocorticoids. In a large cohort study conducted in 2016, nearly two-thirds of patients had notable symptomatic improvement with the use of glucocorticoids or sulfasalazine, whereas refractory cases responded well to interleukin 1, interleukin 6, and tumor necrosis factor-alpha inhibitors.^4^

Partly due to its rarity, Yao syndrome can be challenging to recognize, and more research into its underlying features is needed. Patients presenting with a sequela of dermatitis with unexplained fevers and GI symptoms should raise the suspicion of an autoinflammatory process, particularly diseases associated with a NOD2 mutation. Additionally, uncharacteristic features should not definitively rule out Yao syndrome—such as pediatric onset or absent or uncharacteristic skin findings—and still warrant a full autoimmune and genetic workup. Here, we present the pediatric case of Yao syndrome with novel cutaneous features, such as folliculitis and inflammatory cystic nodules, which further expands our understanding of Yao syndrome and will aid clinicians in identifying this challenging diagnosis.

## Conflicts of interest

None disclosed.
